# Impact of using cross-over CV and mean for two different lots of assay control on implementation of Westgard rules in chemical diagnostic tests

**DOI:** 10.1016/j.plabm.2025.e00449

**Published:** 2025-01-16

**Authors:** Ayman Mohamed Nabil, Hayat Mirza Alsaif, Muneer Ahmad Aljamaan, Abdullah Abdullah H. Algafly, Rashad Hassan aleid, Raji Ali Helal, Zainab Ali Hussain almutawah, Amani Abdulkareem S. Alzayer, Walaa Ali Hussain Almutawah, Badr Abdullah Motlaq AlKhalaf

**Affiliations:** a Ministry of Health, Unnamed Road, النخيل, الرياض, 12382, Saudi Arabia; b Dammam Regional Laboratory, C3JM+73W, Ghirnatah, Dammam, 32245, Saudi Arabia; c Anak General Hospital, 7251 شارع القسمة،, Al Mahar، 3247, Anak, 32455&nbsp, Saudi Arabia; d Branch of the Ministry of Health in the Eastern Province, Saudi Arabia; e Eradah Hospital and Mental Health, King Abdul Aziz Rd, Jazan, Saudi Arabia; f King Faisal Hospital in Al-ahsa, Alfaisaliyah 1st, Al Hofuf, 36361, Saudi Arabia; g Ministry of Health, Dammam, Eastern Region, Kingdom of Saudi Arabia; h Qassim Medical Complex, Saudi Arabia

**Keywords:** *Quality control*, *Westgard rule*, *Cross-over coefficient*, *Accuracy*, *Diagnostic test reliability*, *Reagent lot variation*

## Abstract

**Background:**

The main challenges of clinical laboratories concerning quality control include cost-effectiveness, variability in standardized materials, and evolving technologies across various diagnostic fields. While traditional QC practices and automation systems provide for accuracy, gaps exist, especially when applying Westgard rules to control lots for multiple assays. Such gaps result in inconsistent QC outcomes and unaddressed challenges in diagnostic reliability.

**Objective:**

This study aims to assess the effect of the cross-over coefficient of variation (CV) and mean values for different assay control lots on implementing Westgard rules to improve QC practices and enhance the accuracy and reliability of diagnostic tests in molecular laboratories.

**Methods:**

Data from 18 Levy-Jennings charts, with two assay control lots, were analyzed. Statistical comparisons of failure rates before and after setting the actual SD were performed using chi-square or T-tests at p < 0.05.

**Results:**

The analysis of 18 Levy-Jennings charts showed a significant reduction in failure rates after establishing actual mean and SD values compared to cross-over CV. Of the charts, 11 exhibited differences in failure occurrences, particularly rejection failures, highlighting improved QC reliability.

**Conclusion:**

These results emphasize the importance of accurate SD calculation in enhancing the effectiveness of Westgard rules. Therefore, establishing mean and SD values enhances QC reliability, reduces false failures, and ensures accurate Westgard rules application, while ongoing training in QC practices enhances diagnostic accuracy.

## Highlights

• Assessed the impact of cross-over coefficient of variation and true mean values on improving QC reliability using Westgard rules.
• Analyzed 18 Levy-Jennings charts, revealing a significant reduction in failure rates after applying actual SD and mean values.
• Improved QC reliability through accurate SD calculation, reducing false failures and enhancing diagnostic outcomes.
• Emphasized the need for QC training to ensure precise application of Westgard rules and improved diagnostic accuracy.

## 1 Introduction

Quality Control (QC) in clinical laboratories encounters many challenges, particularly regarding the cost-effectiveness of new and rapidly evolving technologies, especially for high expectations of accuracy for once-in-a-lifetime genetic tests [1,2]. These challenges extend to the lack of quality control materials, standardized calibrators, quantitative test system outputs, tracking software, and the almost daily appearance of new test targets. The term 'cross-over' in this context refers to a transitional approach where data from two assay control lots are pooled to estimate variability in test performance. This method is used when transitioning from one lot to another, allowing for continuity in quality control assessment during the overlap period. Moreover, clinical laboratories struggle to develop appropriate quality assurance programs for molecular diagnostic tests, which are becoming increasingly critical in patient care [3].

In most laboratories, QC programs are mostly automated through laboratory information systems (LIS), which capture and process control data through online integration or manual entry [4]. These QC programs are incorporated in instrument systems and some Point-of-Care devices with similar capabilities. Standalone QC programs on personal computers are also available and offer complete support for calculations, graphic displays of control charts, and results storage [5]. Participants in external survey programs offered by instrument or control manufacturers can submit control data for vendor analysis. However, it may take up to a month for the results of this analysis to be returned. The best practice is to establish the mean and standard deviation (SD) over a 20-day period for each level of control. During the first 20 days of using a new control, the mean and standard deviation (SD) will be pending. In the meantime, the data provided in the package insert will be crucial [6]. Manufacturer-provided values, typically included in the package insert, are recommended for use during the initial setup phase, which generally spans 20–30 days. This period allows sufficient data collection to calculate laboratory-specific mean and SD values. It is essential to transition to laboratory-specific targets as soon as adequate data are available to ensure more accurate quality control limits. When using an assayed control, the package insert typically includes a data sheet, which is either digital or paper, that provides expected or target means and standard deviations. However, without other information, the mean and SD should be set up based on the values provided in the package insert [7].

Hence, the package insert SD typically shows wider ranges than the laboratory SD, often much more significant [8]. Typically, such means are constructed from multiple instruments or even laboratories. Hence, the package insert SD is typically more extraordinary than the laboratory SD, often much more significant. It is preferable to use the laboratory's standard deviation (SD) as soon as possible. The longer the package insert SD is used, the greater the risk of setting control limits that are too wide, which may lead to missing significant errors in test results [9]. Once sufficient data representing your control material's performance is collected from your method and instrument, it is important to begin using that data. Best practices dictate setting up each method and control level with its mean and SD [3].

Moreover, running several controls over a shorter period is advisable to enhance the process of getting an accurate mean and SD [10]. This can be achieved by running four control runs daily for five days. The estimates obtained during the initial runs may be helpful but must be updated with data collected over a more extended period, such as one month. If moving to a new lot of control material, but there is a history for the previous lot, historical mean, coefficient of variation (CV), and SD may be used provisionally for the new lot, with the assumption that the lots are manufactured to produce comparable performance. Nonetheless, at least 20 days of data should be used whenever possible to create new control limits. Because control and reagent lots are never identical, most differences between them are expected to be trivial and have little clinical consequence. The laboratory has to decide what constitutes a clinically significant difference [11].

Control results should have at least one more significant figure than the values reported for patient test results to get reasonable estimates of the mean and SD while maintaining the appropriate control limits. In some instrument systems where test results are rounded for clinical significance, only whole numbers are reported for control results, thereby giving a discrete distribution of control values with only a few possible results, rather than the expected continuous Gaussian distribution. As a result, setting control limits can become problematic, as the calculated limits may not align with the discrete integer values being reported [9,12].

These traditional QC practices, which comply with the best practice and Clinical Laboratory Improvement Amendments (CLIA) regulations, are essential for the ensured accuracy and reliability of the patient's test results [13]. The complexity of Westgard rules, as described by Steindal, poses a significant challenge for clinical laboratories due to their intricate decision-making processes, which require precise calculations and a deep understanding of QC principles. Despite their critical role in improving test reliability, the 2017 Great Global QC Survey conducted by Westgard S revealed that only a fraction of laboratories worldwide consistently applies these rules in practice, indicating a gap in global standardization and accessibility [14]. Specifically, CLIA Sections mandate that laboratories provide written QC procedures for the monitoring and evaluating of each method's analytical testing process in terms of quality. Such critical procedures enable the detection of immediate errors from the system failure in the test process, adverse environmental conditions, and poor operator performance, besides monitoring long-term accuracy and precision for test results. Moreover, molecular diagnostic QC practices should ensure that test components, including extraction steps, are applicable and can accurately reflect shifts or trends in QC results [15].

Despite these established practices, significant gaps in the application of Westgard rules exist, especially on issues related to applying two separate lots of assay controls in molecular diagnostics. Thus, the challenge posed by retesting, low-test production in one lot, and QC results applied to routine Levy-Jennings charts were not adequately addressed. Therefore, this study aims to evaluate the effect of cross-over CV and mean values for two different lots of assay controls on the practical implementation of Westgard rules in chemical diagnostic tests. The goal is to determine how these methods can improve QC practices and ensure more reliable test results in molecular laboratories.

## 2 Methodology

This study aimed to assess the effects of cross-over CV in determining the SD and setting actual mean and SD values to failure rates concerning the standard Westgard rules. Data from 18 Levy-Jennings charts was used to analyze and monitor the performance of chemical diagnostic tests. For each test system, two different lots of assay controls were utilized to check the variability and credibility of testing. Additionally, the potential impact of reagent lot variation on QC performance was considered. This study recommends conducting independent validation of new reagent lots by performing side-by-side comparisons with previous lots. Such practices can identify significant lot-to-lot variations early and mitigate their impact on QC reliability. Some of these control materials were taken to determine routine quality-control tests and check the efficiency and reliability of the testimonial system.

Initially, the SD was calculated using the cross-over CV method by pooling data from both assay control lots to estimate variability in test performance. Then, the calculated SD was compared using the cross-over CV method with the actual mean, and SD values were established later for each control lot. After determining the actual mean and SD values, control results were plotted on Levy-Jennings charts, and control limits were defined accordingly. However, traditional Westgard rules were applied to the control data to identify failures, warning failures, and rejection failures for each of the 18 charts, while the failures were considered when a data point fell outside the control limits defined by the Westgard rules. In the end, the number of failures was recorded for both conditions, such as before and after establishing the actual mean and SD values. This methodology allowed for a comparison of failure rates under both conditions.

Comparisons between the failure rates before and after the changes occurred with the implementation of actual mean and SD values were made using an inferential statistical method. The total failures, warning failures, and rejection failures made during the experiment were counted and compared between the two conditions. Fisher exact or chi-square test and T-test were used when appropriate, at a p < 0.05 significance level. The occurrence directions of all failures were compared and contrasted according to this threshold level.

## 3 Results

The QC results were plotted on Levy-Jennings charts to monitor failures according to the "Westgard Rules." The failures were compared before and after establishing the mean and standard deviation (SD). Initially, the SD was calculated using cross-over cross-validation (CV), and after establishing the actual mean and SD, the failures were monitored again as an example of adopting these values.

Table 1 below summarises the number of failures, warning failures, and rejection failures before and after establishing the actual mean and SD for each of the 18 test groups.

**Table 1:** Number of failures according to traditional "Westgard rules".

Group No	Before establishment of actual mean and standard deviation using cross-over CV to calculate the SD			After the establishments of actual mean and standard deviation, a			Difference Yes or No
	Number of total failures occurred	Number of warning failure occurred	Number of Rejection failure occurred	Number of total failure occurred	Number of warning failure occurred	Number of Rejection failure occurred	
**1**	0	0	0	0	0	0	No
**2**	0	0	0	1	1	0	Yes
**3**	3	3	0	3	0	3	Yes
**4**	1	1	0	0	0	0	Yes
**5**	0	0	0	0	0	0	No
**6**	5	3	2	1	1	0	Yes
**7**	3	2	1	8	1	7	Yes
**8**	2	1	1	1	0	1	Yes
**9**	3	2	1	2	2	0	Yes
**10**	1	1	0	1	1	0	No
**11**	2	2	0	8	3	5	Yes
**12**	0	0	0	0	0	0	No
**13**	1	1	0	1	1	0	No
**14**	1	1	0	0	0	0	Yes
**15**	0	0	0	0	0	0	No
**16**	1	1	0	1	0	1	Yes
**17**	2	2	0	2	0	2	No
**18**	1	1	0	7	2	5	Yes
**Total**	**26**	**21**	**5**	**36**	**12**	**24**	**Yes**

The 18 Levy-Jennings charts were analyzed, and differences in failure occurrences were found before and after establishing the mean and SD values. Of the 18 charts, 7 were comparable in failure rates, while 11 had differences in failure occurrences when comparing the two conditions. Specifically, 8 charts recorded rejection findings for rejection failures, with only 1 chart showing comparable results between the two conditions. The remaining 7 charts showed much difference in the number of rejection failure occurrences. Overall, the data indicated that the establishment of the actual mean and SD values had a substantial impact on the failure rates, especially in the case of rejection failures, where most charts showed a noticeable improvement or variation. These findings highlighted a need to accurately determine the mean and SD to reduce the number of false failures, thereby enhancing chemical diagnostic test reliability when utilizing the traditional Westgard rules.

## 4 Discussion

This study evaluated the impact of cross-over CV and real mean and SD on enforcing Westgard rules in chemical diagnostic tests. The significant findings were the qualitative and quantitative differences in the number and distribution of the QC failures when using the traditional Westgard rules with different actual mean and SD values. The qualitative results derived from the given actual mean and SD value were that the number of failures regarding the point-cross over CV for estimating SD was higher and more specifically to the rejection failures than the number as provided … The findings presented here provide compelling arguments that challenge the adequacy of cross-over CV to be the right path for SD estimation in QC procedures.

While the study demonstrates the advantages of laboratory-specific QC target values, it is essential to acknowledge that many laboratories depend on manufacturer-assigned targets due to cost constraints. However, calculating in-house QC target values, though initially resource-intensive, can result in long-term savings by improving diagnostic accuracy and reducing false failures. Laboratories should evaluate the cost-benefit trade-offs and consider adopting this approach as part of their QC practices.

### 4.1 Impact of cross-over CV on SD calculation and Westgard rule interpretation

Multiple investigations have demonstrated that cross-over CV was used for SD estimation, resulting from the question mark regarding the reliability of CV representation of variability [16,17]. These concerns are also in line with the findings of this study because differences in the cross-over CV caused dissimilar failure rates like missed warning or rejection failure. The samples below or above CLI stated the possible error in the test system, for instance, instrument failure, human error, or environmental influence, as per Westgard rules [18]. Conversely, when cross-over CV was employed to estimate SD, the failure rates were underreported, which might cover up some inefficiency in the testing process [19].

Some authors have noted that accurate estimates of SD are critical when applying Westgard rules. Moreover, a lack of accurate SD values could lead to wrong control limits, where the control charts might generate false positive or negative results [20]. However, our data analysis confirms the possibility of misinterpreting the quality control charts when applying the cross-over CV to calculate SD and, therefore, distorting the results of the diagnostic test.

### 4.2 The importance of establishing actual mean and SD values

The use of small sample sizes, such as 20 samples, for initial QC target calculations can introduce variability and potential errors in establishing reliable QC limits. To minimize such risks, laboratories should collect a larger dataset over an extended period and periodically recalibrate the QC targets. This ensures more accurate representation of assay performance and reduces the likelihood of diagnostic errors.

It is crucial to emphasize that the problem lies in the reliance on outdated or inaccurate mean and SD calculations. These values must be regularly reviewed and updated—ideally on a monthly basis—to ensure they accurately reflect assay performance and account for any variability. Regular recalibration of QC targets is essential to maintaining reliable diagnostic outcomes and minimizing false failures.

Based on the findings of this study, it is recommended that true mean and SD values be set to enhance the efficiency of the QC monitoring. In this way, these values and constants get calculated, resulting in more accurate control charts and Westgard rules being more effective and constructive. Furthermore, it was demonstrated in this study that by appealing to the correct mean and SD values that would represent the particular sample, the failure rates should be compared before and after the formulation of the actual mean and SD values. This approach prevents such situations as missing failures and contributes to the higher accuracy of the quality examination in diagnostic laboratories.

Past research has indicated that if laboratories employ other general or wrong methods to determine SD, for example, cross-over CV, there can be negative impacts on the quality of testing results. For example, according to Ellis et al. (2020), the CV in quality control data varies significantly among QC and reagent lots and between identical specification instruments, affecting uncertainty estimation and interpretation of QC results [21]. Consequently, according to Brooks (2024), best practice for quality control involves putting a meaningful recent mean and SD on the QC chart, selecting rules based on the method's margin for error to unacceptable risk or Sigma value, and basing acceptable risk on clinical need [22].

### 4.3 Practical implications for QC practices

The findings of this study are highly significant for the contemporary practices of QC in clinical laboratories. The findings underscore the importance for laboratories to verify that suitable procedure for SD estimation exists primarily when in Westgard rules to assess the performance of the test. Despite its application, cross-over CV may yield a quick approximation of the variability but does not consider the pattern and performance of the different assay lots, resulting in poor SD determination and ineffective QC assessment.

Based on the risks detected in the current work, laboratories should employ more accurate methods of SD calculation, as well as actual mean and SD values for each lot. Furthermore, reagent lot variation poses a critical challenge to consistent QC performance. Laboratories are advised to independently assess new reagent lots by comparing them against established performance benchmarks before routine implementation. This practice not only ensures continuity in QC but also reduces the risk of diagnostic inaccuracies caused by lot-to-lot variability. The above-formulated methods facilitate improved control chart analysis and better identification of QC failures. In addition, progressive training and education on the technique involved and applying Westgard rules and SD estimation methods are also required to enhance quality control practices and reduce the wrong interpretations of clinical tests.

## 5 Conclusion

This study emphasizes using mean and standard deviation values when applying Westgard rules for QC in chemical diagnostic tests. Actual mean and SD values were more reliable than cross-over CV methods in producing QC results, reducing false failures while improving the detection of actual failures, especially rejection failures. Implementing precise control limits ensures reliable diagnostic tests with consistent performance. To ensure the continued reliability of QC processes, laboratories must regularly recalibrate QC target values, especially when assay variability or reagent lot changes are observed. A systematic recalibration schedule, such as quarterly reviews, can help maintain the robustness of diagnostic procedures. Strong methodologies and continuous education on QC practices are highly recommended to retain accuracy and build confidence in clinical outcomes in delivering patient care and reliable diagnosis.

## 6 Limitations and future directions

This study had several limitations, such as being focused on a small dataset of 18 Levy-Jennings charts and variability in assay control lots among different laboratories. Also, generalizing the results may be restricted by different practices in SD estimation and QC methods. Further research should be conducted using more extensive data sets and different test systems to validate these findings further. Automated methods for calculating mean and SD could also be explored to simplify QC processes. Combining machine learning to predict QC failures and optimize Westgard rule applications may further enhance diagnostic accuracy and laboratory productivity.

## CRediT authorship contribution statement

**Ayman Mohamed Nabil:** Formal analysis, Data curation, Conceptualization. **Hayat Mirza Alsaif:** Methodology, Investigation, Formal analysis. **Muneer Ahmad Aljamaan:** Project administration, Methodology. **Abdullah Abdullah H. Algafly:** Validation, Supervision, Software. **Rashad Hassan aleid:** Supervision, Software. **Raji Ali Helal:** Data curation, Conceptualization. **Zainab Ali Hussain almutawah:** Methodology, Investigation. **Amani Abdulkareem S. Alzayer:** Visualization, Validation, Supervision. **Walaa Ali Hussain Almutawah:** Formal analysis, Data curation. **Badr Abdullah Motlaq AlKhalaf:** Resources, Project administration, Conceptualization.

## 7 Authorship

All authors have made substantial contributions to the conception and design of the study, acquisition, analysis, and interpretation of the data. Each author has participated in drafting the article or revising it critically for important intellectual content and has given final approval of the version to be submitted. All authors agree to be accountable for all aspects of the work, ensuring questions related to the accuracy or integrity of any part of the manuscript are appropriately investigated and resolved.

## Acknowledgment of inclusive language

The authors have adhered to guidelines on inclusive language, ensuring the manuscript respects diversity and conveys sensitivity. Efforts have been made to maintain neutrality and avoid bias in terminology.

## Use of generative AI

During the preparation of this work, the authors used AI tools (e.g., language models) only to enhance the clarity and readability of the manuscript. All content has been reviewed and verified by the authors to ensure accuracy and integrity. The authors take full responsibility for the final content.

## Funding sources

This research did not receive any specific grant from funding agencies in the public, commercial, or not-for-profit sectors.

## Declaration of competing interest

The authors declare the following financial interests/personal relationships which may be considered as potential competing interests: Ayman Mohamed Nabil reports statistical analysis was provided by Saudi Arabia Ministry of Health. Ayman Mohamed Nabil reports a relationship with Saudi Arabia Ministry of Health that includes: non-financial support. Ayman Mohamed Nabil has patent pending to None. None If there are other authors, they declare that they have no known competing financial interests or personal relationships that could have appeared to influence the work reported in this paper.

## Data Availability

No data was used for the research described in the article.
